# Polymorphism of the Melanocortin 1 Receptor (*MC1R*) Gene and its Role in Determining the Coat Colour of Central European Cattle Breeds

**DOI:** 10.3390/ani10101878

**Published:** 2020-10-14

**Authors:** Karolina Kasprzak-Filipek, Wioletta Sawicka-Zugaj, Zygmunt Litwińczuk, Witold Chabuz, Rūta Šveistienė, Josef Bulla

**Affiliations:** 1 Sub-Department of Cattle Breeding and Genetic Resources Conservation, Institute of Animal Breeding and Biodiversity Conservation, University of Life Sciences in Lublin, Akademicka 13, 20-950 Lublin, Poland; karolina.kasprzak-filipek@up.lublin.pl (K.K.-F.); zygmunt.litwinczuk@up.lublin.pl (Z.L.); witold.chabuz@up.lublin.pl (W.C.); 2 Animal Science Institute, Lithuanian University of Health Sciences, A. Mickeviciaus 9, LT 44307 Kaunas, Lithuania; ruta.sveistiene@lsmuni.lt; 3 Department of Animal Physiology, Slovak University of Agriculture in Nitra, A. Hlinku 2, 94976 Nitra, Nitriansky Kraj, Slovakia; bulla@uniag.sk

**Keywords:** *MC1R*, coat colour determination, Central European cattle breeds

## Abstract

**Simple Summary:**

Animal coat colour has been the subject of numerous studies for many years. While most phenotypic features of animals result from the interaction of genetic and environmental factors, coat colour is considered to be almost exclusively genetically determined. Differences in coat colour underscore the distinct character of a given breed or group of animals, testify to its uniqueness, and sometimes serve as selection criteria. Observations of changes in cattle coat colour are an important source of information used to track domestication processes and discover how animals were selected for breeding. One of the genes responsible for variation in coat colour is the melanocortin receptor (*MC1R*) gene locus, which controls the production of black and red pigments that determine basic colours. Other interacting genes also influence cattle phenotypes. In view of the complexity of the genetic factors influencing cattle coat colour, this study investigated the genetic basis of different coat colours of Central European cattle breeds.

**Abstract:**

There are many genes responsible for the appearance of different coat colours, among which the melanocortin 1 receptor gene (*MC1R*) plays an important role. The aim of the study was to characterize genetic variation in Central European cattle breeds based on polymorphism of the *MC1R* gene and factors determining their coat colour. The study was conducted on 290 individuals of the following breeds: Polish White-Backed (PW), Lithuanian White-Backed (LW), Polish Red (PR), Lithuanian Red (LR), Carpathian Brown (CB), Ukrainian Grey (UG), and Slovak Pinzgau (SP). Polymorphism at the *MC1R* gene locus was analysed by polymerase chain reaction-restriction fragment length polymorphism (PCR-RFLP) using two restriction enzymes: *Cfr10I* and *SsiI*. The proportions of alleles and genotypes in the *MC1R* locus indicates a strong relationship between polymorphism and the coat colour of cattle: The E^D^ allele proved to be characteristic for the breeds with a white-backed coat (PW and LW), while the dominant allele in the red breeds (PR and LR) was E^+^. It is noteworthy that coat colour in the SP population was determined only by the recessive e allele, which resulted in the formation of a separate clade in the phylogenetic tree.

## 1. Introduction

A characteristic feature of most phenotypic traits of animals is that they are the result of the interaction of genetic and environmental factors. The coat colour of mammals depends primarily on the proportions of individual pigments: Pheomelanin (red pigment) and eumelanin (determining brown or black colour) [1,2]. The proportion of one of the two pigments is limited by tyrosinase, an enzyme that limits their synthesis. A low tyrosinase level leads to the production of pheomelanin, while increased tyrosinase production results in the production of eumelanin [3]. Two loci are primarily responsible for regulating the level of pigments produced by melanocytes: E (Extension) and A (Agouti) [4]. These loci exert an epistatic effect in various mammalian species. Alleles dominant at the E locus determine the colour black (eumelanin production), while recessive alleles, due to pheomelanin synthesis, cause the appearance of a colour from red to pale yellow. Mutations occurring at the Agouti locus have the opposite effect: Dominant alleles determine pheomelanin production, while recessive ones are usually associated with eumelanin synthesis and black colour [5].

The E locus encodes the melanocortin 1 receptor (*MC1R*), which binds to melanocyte-stimulating hormone (*α-MSH*), which induces eumelanin synthesis [6,7]. The A locus, which encodes the protein *ASIP* (agouti-signalling protein), by acting antagonistically to the E locus causes blockage of α-*MSH* receptor interaction, resulting in the production of pheomelanin instead of eumelanin [8,9].

Mutation at the *MC1R* gene locus has been the subject of many studies on various species of mammals, such as humans [10], pigs [11], felids [12], mice [13], sheep [14], dogs [15], foxes [16], bears [17], horses [18] and cattle [3,19,20], where the occurrence of a functional mutation has been associated with black (or dark) coat colour, while a lack of mutation at the *MC1R* locus has resulted in red, yellow or white colours.

The *MC1R* gene in cattle is located on chromosome 18 [3]. According to research in cattle conducted by Adalsteinsson et al. [21], the colours black, brown and red are determined by the occurrence of alleles from the E and A loci. Three alleles have been distinguished at the E locus in order to clarify the mechanism of inheritance of coat colour in cattle: The dominant black E^D^ allele, the recessive red e allele, and the E^+^ allele, which enables phenotypic expression of the A locus alleles. Individuals with the E^+^ allele probably have the A^+^ allele responsible for the colour brown or the recessive allele a determining the colour black at the A locus. The order of allele dominance is usually described as E^D^ > E^+^ > e. Analyses carried out by Klungland et al. [3] have shown that information obtained by sequencing or other methods e.g., restriction fragment length polymorphism (RFLP) can be used to identify three alleles at the *MC1R* gene locus.

The aim of the study was to characterise genetic variation in Central European cattle breeds based on polymorphism of the *MC1R* gene and factors determining their coat colour.

## 2. Materials and Methods

### 2.1. Study Area, Data Collection and Characteristics of Populations Selected for Research

The animals came from private farms located in Poland, Lithuania, Ukraine and Slovakia. The study was conducted on 290 individuals belonging to seven Central European cattle breeds kept on 57 farms. The animals were not related, which was verified on the basis of breeding documentation and pedigree data (to two generations back). Hair bulbs were used as biological material.

**Figure 1:**
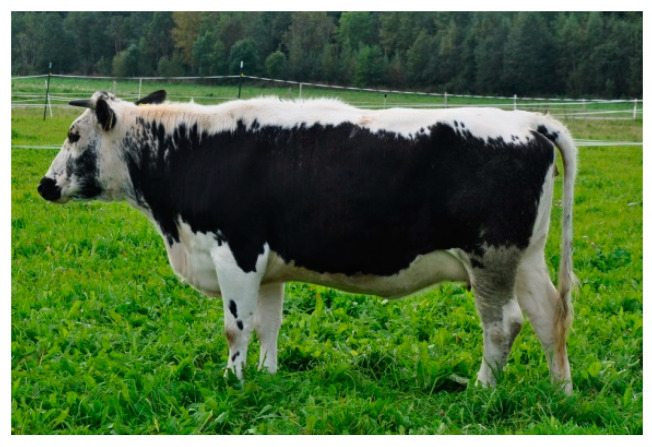
Polish White-Backed (PW) female.

Polish White-Backed cattle (PW) were raised in eastern Poland, in a temperate climate. The animals had black sides and a continuous stripe running from the muzzle, which was dark, along the entire body (Figure 1). A total of 50 animals from 10 farms were selected for the study.

**Figure 2:**
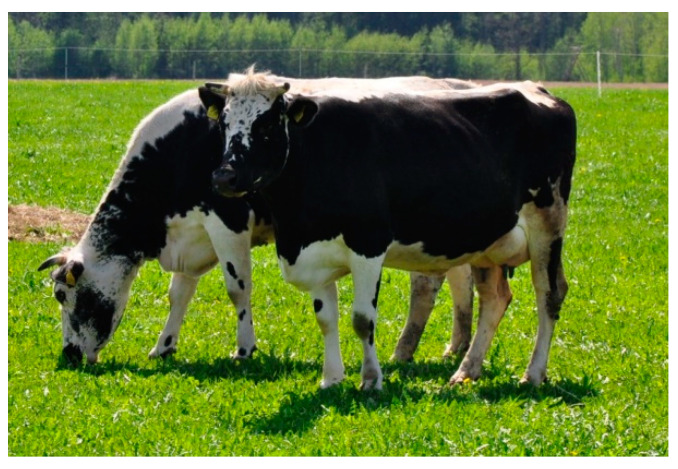
Lithuanian White-Backed (LW) females.

Lithuanian White-Backed cattle (LW) were raised in central Lithuania, in a temperate climate. The animals had black sides and a continuous stripe running from the muzzle, which was dark, along the entire body (Figure 2). A total of 50 animals from 10 farms were selected for the study.

**Figure 3:**
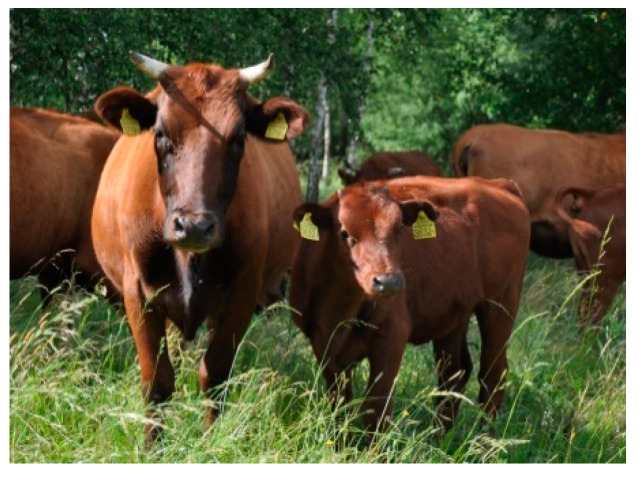
Polish Red (PR) female with calf.

Polish Red cattle (PR) were raised in Southern Poland, in a temperate climate. The animals had a solid red coat and a dark muzzle (Figure 3). A total of 50 animals from 10 farms were selected for the study.

**Figure 4:**
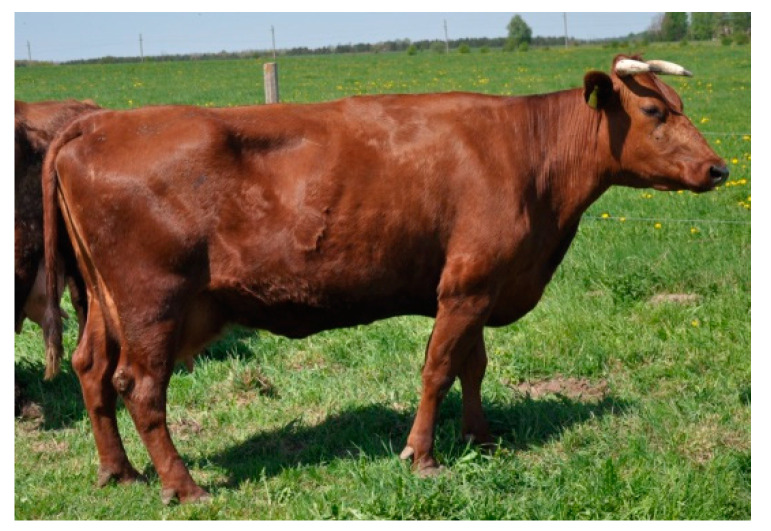
Lithuanian Red (LR) female.

Lithuanian Red cattle (LR) were present in central Lithuania, in a temperate climate. The animals had a solid red coat and a dark muzzle (Figure 4). A total of 50 animals from 10 farms were selected for the study.

**Figure 5:**
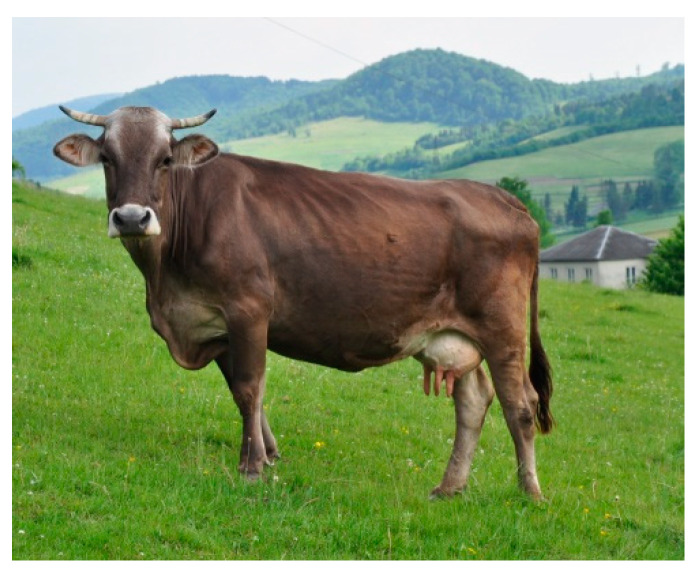
Carpathian Brown (CB) female.

Carpathian Brown cattle (CB) were raised in Western Ukraine (Zakarpattia), in a transitional temperate climate (influenced by continental climate). The animals had a solid coat colour, from dark brown to light grey, with a characteristic white ring around the nostrils (Figure 5). A total of 22 animals from 5 farms were selected for the study.

**Figure 6:**
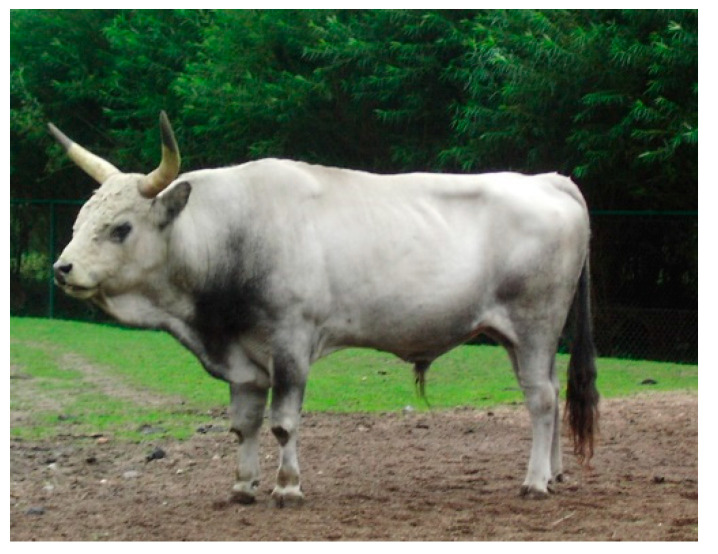
Ukrainian Grey (UG) male.

Ukrainian Grey cattle (UG) were raised in Eastern Ukraine (steppe region), in a continental climate (hot summer and freezing winter). The animals had a solid grey or light grey coat colour, often with a darker shade on the neck, chest or abdomen (Figure 6). A total of 18 animals from five farms were selected for the study.

**Figure 7:**
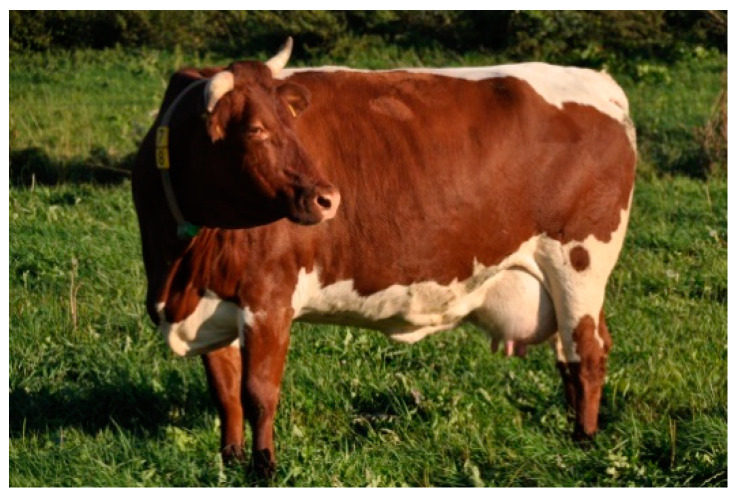
Slovak Pinzgau (SP) female.

Slovak Pinzgau cattle (SP) were raised in Northern Slovakia, in a cool temperate climate. The animals had a red coat with a white stripe running from the withers and crossing the tail, udder and underbelly, and a solid red head (Figure 7). A total of 50 animals from 7 farms were selected for the study.

### 2.2. Molecular Identification

The genomic DNA of cattle was isolated using a ready-made commercial kit for nucleic acid isolation from biological traces (Sherlock AX A&A Biotechnology, Gdynia, Poland) according to the procedure provided by the manufacturer. Polymerase chain reaction-restriction fragment length polymorphism (PCR-RFLP) was used to analyse polymorphisms at the *MC1R* locus in the seven Central European cattle breeds.

Primers given by Klungland et al. [3] were used to assess polymorphism at the *MC1R* gene locus. The first primer pair was 5′-GTGCCTGGAGGTGTCCATC-3′ (forward primer) and 5′-GAAGTTCTTGAAGATGCAGCC-3′ (reverse primer), and the second primer pair was 5′-CAAGAACCGCAACCTGCACT-3′ (forward primer) and 5′-GCCTGGGTGGCCAGGACA-3′ (reverse primer). PCR-RFLP was used to amplify the *MC1R* gene fragment.

The amplified fragments were digested with Thermo Scientific FastDigest™ (Thermo Fisher Scientific, Waltham, MA, USA) restriction enzymes *Cfr10I* and *SsiI*. The digestion reaction was carried out at 37 °C.

The fragments obtained from the PCR-RFLP reaction were subjected to electrophoretic separation on a 2% agarose gel stained with 0.01% ethidium bromide (EtBr). Molecular size markers (Thermo Scientific GeneRuler 100bp Plus DNA, Thermo Fisher Scientific) were used to track the electrophoresis process and assess the length of the fragments.

### 2.3. Statistical Analysis

To visualize the genetic diversity of the analysed cattle breeds, the results were subjected to statistical analysis in POPGENE v. 3.2. statistical software. The following indicators were estimated: Frequency of alleles and genotypes, observed heterozygosity (H_O_) and genetic distances according to Nei [22]. The obtained values of genetic distances were used to create a phylogenetic tree using UPGMA (unweighted pair group method with arithmetic mean) method.

## 3. Results

**Figure 8:**
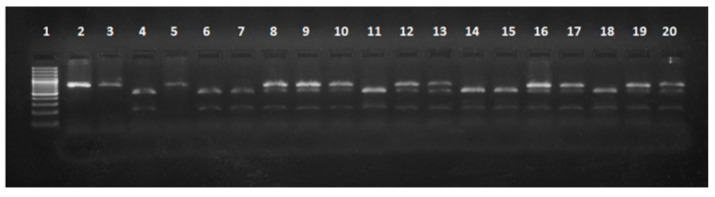
Electrophoretic separation of DNA fragments at the melanocortin 1 receptor (*MC1R*) gene locus following digestion with restriction enzyme *Cfr10I*: lane 1: DNA ladder, lane 2: PCR (polymerase chain reaction product—739 bp), Identyfied genotypes: lanes 8–10,12,13,16,17,19,20: ED/e, E+/e (739 bp, 531 bp, 208 bp), lanes 3,5: ee (739 bp), lanes 4,6,7,11,14,15,18: ED/ED, ED/E+, E+/E+ (531 bp, 208 bp).

Identification of genotypes at the *MC1R* locus was carried out in two stages. Restriction digestion of the PCR product (739 bp) with the *Cfr10I* enzyme resulted in genetic variants E^D^/e and E^+^/e, where the lengths of the fragments were 739 bp, 531 bp and 208 bp; ee with a length of 739 bp; and E^D^/E^D^, E^D^/E^+^ and E^+^/E^+^, with fragments of 531 bp and 208 bp (Figure 8).

**Figure 9:**
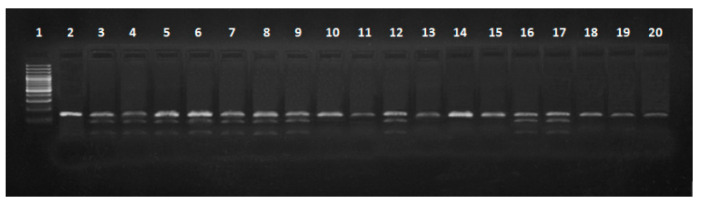
Electrophoretic separation of DNA fragments at the melanocortin 1 receptor (*MC1R*) gene locus following digestion with restriction enzyme *SsiI*: lane 1: DNA ladder, lane 2: PCR (polymerase chain reaction product—130 bp), lanes 3–9,12,16,17: E^D^/E^+^, E^D^/e (130 bp, 97 bp, 33 bp), lane 10,11,13–15, 18–20: E^+^/E^+^, E^+^/e, e/e (130 bp).

In the second stage of the analysis, the amplification resulted in a 130 bp product. Following restriction digestion of the PCR product with the *SsiI* enzyme, the following genotypes were obtained: E^D^/E^+^ and E^D^/e, with fragments of 130 bp, 97 bp and 33 bp; and E^+^/E^+^, E^+^/e and e/e, where cleavage of the products did not take place (Figure 9).

The analyses performed at the *MC1R* gene locus identified five genotypes, E^D^/e, E^D^/E^+^, E^+^/e, E^+^/E^+^ and e/e, determined by the occurrence of three alleles: The dominant E^D^ allele, the recessive e allele, and the wild type E^+^ allele. The tables present the percentages of individuals in each breed for which the genotypes were identified at the *MC1R* locus (Table 1) and the frequencies of alleles at the *MC1R* locus for the breeds (Table 2).

**Figure 10:**
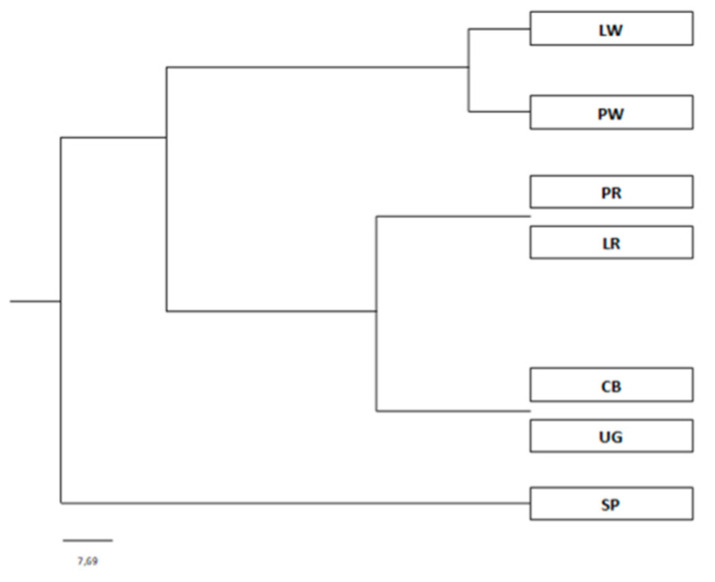
Dendrogram of genetic similarity for seven Central European cattle breeds according to the UPGMA (unweighted pair group method with arithmetic mean) method, based on genetic similarity calculated according to Nei’s formula [22]. Notation: LW—Lithuanian White-Backed, PW—Polish White-Backed, PR—Polish Red, LR—Lithuanian Red, CB—Carpathian Brown, UG—Ukrainian Grey, SP—Slovak Pinzgau.

Coat colour in cattle is determined by a number of genes, among which an important role is attributed to the *MC1R* gene. Our research shows that assessment of polymorphism at the *MC1R* gene loci makes it possible to partially explain determination of coat colour, and at the same time is an important source of information in the analysis of the genetic diversity of Central European cattle breeds. The phylogenetic tree (Figure 10), based on estimated values of distances and genetic similarities according to Nei [22], shows that the Central European cattle breeds included in the study formed three distinct clades, according to their coat colour characteristics. The first clade included both breeds representing the White-Backed coat type (with black sides), i.e., PW and LW. White-Backed cattle are derived from primitive cattle originally present in north-western Europe. Cattle with this coat colour became widespread in the Baltic Sea region together with German settlers during the Middle Ages [23,24]. The second clade comprised two groups of single-colour breeds—red-coated breeds (PR and LR) and grey and brown cattle (UG and CB). These are Central-Eastern European breeds. PR and LR are derived from small, wild short-horned cattle living in Eastern Europe. The spread of these animals is associated with the migration of Slavic peoples in the early 16th century [25]. The UG and CB breeds, which were in one group, had a common link—Podolian cattle, which were used in their creation. UG is a breed derived from Podolian Grey Steppe cattle kept in Romania, Hungary, Italy and Bulgaria in the 19th century [26]. The CB breed was probably created through many years of cross-breeding of local brown breeds with imported Alpine and Podolian breeds [27]. SP cattle formed a completely separate clade, which reflects the distinct differences in both the colour and location of the breed relative to the others. This breed is included among Alpine (Central European) cattle [28]. According to the estimated genetic distances, the highest genetic similarity was between the CB and UG breeds, while the highest genetic diversity estimated on the basis of polymorphism analysis at the *MC1R* gene locus was found between the LR and SP breeds.

## 4. Discussion

The dominant E^D^ allele was identified in the PW and LW breeds. Given the characteristic black and white colour of the White-Backed individuals included in the study, the results obtained are consistent with those reported by Russo et al. [29], who identified the E^D^ allele in the Italian Holstein–Friesian and Black Pied Valdostana breeds; by Rouzaud et al. [20], who found the E^D^ allele to be characteristic of the French Holstein–Friesian breed; and by other authors [19,30,31,32].

The wild type E^+^ allele was present with varying frequency in six analysed breeds, but was not identified in the SP breed (Table 2). Importantly, individuals belonging to different Central European cattle breeds, and thus differing in coat colour (from the light-grey UG to the red PR and LR breeds), were found to have the E^+^ allele. According to Klungland et al. [3], the wild type receptor encoded by the E^+^ allele is probably inhibited, so that the dominant A locus inhibits the *MC1R* locus effect, resulting in different coat colours in cattle. Similar results have been reported by other authors [20,29,33], who found that the E^+^ allele determined differences in coat colour. Rouzaud et al. [20] identified the E^+^ allele in individuals of the Gascon breed with a grey coat, in Normande cattle with a brown and white coat, and in brown Aubrac individuals. A study by Lee et al. [33] analysing brown, yellow-brown, and black individuals of Korean and Japanese breeds, also identified individuals with the wild type E^+^ allele. Similarly, in a study by Russo et al. [29], the E^+^ allele was identified among specimens of numerous breeds with a variety of coat colours: Modenese, Jersey, Simmental, Grigio Alpina, Piedmontese, Chianina, Romagnola, Marchigiana, Swedish Red and White, and Danish Red. On the other hand, Brenig et al. [34] found that individuals with the E^+^E^+^ genotype at the *MC1R* locus had a red coat colour.

The occurrence of the e allele was recorded among individuals of all breeds tested, but it is significant that all SP individuals had the e/e genotype at the *MC1R* locus. In the study by Russo et al. [29], of the many European (mainly Italian) breeds analysed, only Reggiana individuals had the e/e genotype. Rouzaud et al. [20] found that the coats of individuals with e/e genotypes were very light (Blonde d’Aquitaine), cream-and-white (Charolais), red (Limousin), and dark mahogany (Salers). According to the assumptions of Klungland et al. [3], all individuals with e/e genotypes have a red coat, whereas the results of the authors’ analyses and our own research may indicate a modifying effect of various genes, including the A locus (agouti), on the expression of the *MC1R* gene, thereby causing varied coat colour in different breeds of cattle.

Research by Putra et al. [35] has demonstrated that the environmental conditions in which animals are raised may also have a modifying effect on coat colour, and indirectly on the proportions of alleles at the *MC1R* gene locus. The estimated allele frequencies among Indonesian Pesisir cattle were 0 for E^D^, 0.93 for E^+^ and 0.07 for e. Two genotypes were identified among the individuals studied: E^+^E^+^ and E^+^e. The genotypes E^D^E^D^, E^D^E^+^, E^D^e and ee were not observed. The proportions of the alleles obtained by the authors may be the result of a selective environmental effect, with the light coat of the Indonesian Pesisir cattle (determined by the E^+^ and e alleles at the *MC1R* locus) enabling survival in tropical conditions.

The estimated frequency of alleles and genotypes at the *MC1R* locus and the observed variations in coat colour in the Central European cattle breeds may also be due to their geographic distribution. In a study by Niemi et al. [36], which assessed polymorphism at the *MC1R* locus in native Scandinavian breeds, the phenotypes were strongly influenced by the environment of the region where the animals were kept. High proportions of heterozygotes (from 26% to 57%) were observed among individuals of the native Scandinavian breeds, with both uniform and multi-coloured coats. In contrast, the level of heterozygosity at the *MC1R* gene locus in Western, Central and Southern European breeds was much lower (from 0% to 16%). In our research, the average percentage of heterozygous individuals among all analysed Central European cattle breeds was high, amounting to 53%. The high heterozygosity levels at the *MC1R* gene locus for the PW, LW, UG and CB breeds are likely due to the population bottleneck effect. Previous studies have shown that the current structure of these cattle populations is due to a sudden decline in the number of individuals and its subsequent restoration from the group of surviving individuals [37,38].

According to Mei et al. [39], analysis of polymorphism at the loci of various genes associated with milk traits, meat quality or coat colour (including the *MC1R* gene locus) makes it possible to track domestication processes and identify the characteristics of a given breed. The authors’ research on individuals of Chinese cattle breeds offers a new view of evolutionary history and the features of domestication of Chinese cattle. Ludwig et al. [40] also state that coat colour is a feature that can be used to track the domestication processes. Differences in coat colour underscore their distinct character and testify to the uniqueness of a given breed or group of animals, sometimes serving as a selection criterion [41].

In addition, a comparative analysis by Niemi et al. [36] of ancient DNA of cattle from Finland and contemporary Scandinavian cattle showed that the frequency of *MC1R* alleles exhibits changes over time that are similar to those found in studies of mitochondrial DNA and Y chromosome haplotypes. At the locus of the *MC1R* gene as well as the other two markers there was a significant change in genotypes in Scandinavian cattle from the late Iron Age to the Middle Ages, followed by a period of slower change, continuing up to the present day.

## 5. Conclusions

The results of analyses of polymorphism at the *MC1R* gene locus contribute to knowledge of the genetic variation between Central European cattle breeds, their phylogenetic relationships, and the characteristics of a given breed. The proportions of alleles and genotypes at the *MC1R* locus indicate a strong relationship between polymorphism and the coat colour of cattle. The E^D^ allele was characteristic of the white-backed breeds (PW—0.46 and LW—0.50) but was not present in the other breeds analysed. In the PR and LR breeds, the wild-type allele E^+^ had the largest share (0.90 and 0.94, respectively). The alleles were evenly distributed in the CB and UG breeds, and all individuals were heterozygous (E^+^/e). The e allele occurred in all breeds, with the homozygous e/e genotype found only in the SP breed (100% of the studied population). Given the complexity of the genetic mechanisms responsible for the appearance of varied coat colours, such as the characteristic coat of the White-Backed breeds and the SP breed or the uniform coat colours found in the red breeds and the CB and UG breeds, research should be expanded to include analysis of polymorphism at other loci associated with coat colour in cattle.

## Figures and Tables

**Table 1:** Observed heterozygosity (H_o_) values and percentage shares of individuals with identified genotype at the melanocortin 1 receptor gene *(MC1R)* locus (%).

Breed	Heterozygosity Observed (H_o_)	Genotype in *MC1R* Locus					
		E^D^/e	E^+/^e	E^D^/E^+^	E^D^/E^D^	E^+^/E^+^	e/e
LW	1.00	84%		16%			
PW	0.98	28%	6%	64%		2%	
PR	0.20		20%			80%	
LR	0.12		12%			88%	
CB	1.00		100%				
UG	1.00		100%				
SP	0.00						100%

Notation: LW—Lithuanian White-Backed, PW—Polish White-Backed, PR—Polish Red, LR—Lithuanian Red, CB—Carpathian Brown, UG—Ukrainian Grey, SP—Slovak Pinzgau.

**Table 2:** Frequency of alleles at the melanocortin receptor gene (*MC1R)* locus for the breeds tested.

Alleles	Allele Frequency in Breeds							Overall Frequency
	LW	PW	PR	LR	CB	UG	SP	
***E^D^***	0.50	0.46	-	-	-	-	-	0.17
***E^+^***	0.08	0.37	0.90	0.94	0.50	0.50	-	0.46
***e***	0.42	0.17	0.10	0.06	0.50	0.50	1.00	0.37

Notation: LW—Lithuanian White-Backed, PW—Polish White-Backed, PR—Polish Red, LR—Lithuanian Red, CB—Carpathian Brown, UG—Ukrainian Grey, SP—Slovak Pinzgau.
